# Reframing Functional Neurological Disorders in Modern Medicine

**DOI:** 10.7759/cureus.87415

**Published:** 2025-07-07

**Authors:** Marius P Iordache

**Affiliations:** 1 Faculty of Medicine, Titu Maiorescu University, Bucharest, ROU; 2 Clinical Neurology, Ilfov County Clinical Emergency Hospital, Bucharest, ROU

**Keywords:** functional disorder, functional neurological disorder, multidisciplinary approach, neurology and psychiatry, stigma in health care

## Abstract

Functional neurological disorders (FNDs) present a unique intersection between neurology and psychiatry, encompassing a range of neurological symptoms not attributable to structural neurological diseases, often challenging traditional diagnostic and therapeutic approaches. Recent advancements have shed light on its epidemiology, pathophysiology, and management. This editorial reframes FND in light of recent advances, highlighting how evolving understanding, epidemiological insights, and emerging treatments are reshaping its status in global healthcare. The aim is to foster an integrated narrative: FND is no longer a diagnosis of exclusion but a diagnosable and potentially treatable disorder. This reframing promotes more accurate diagnosis and integrated treatment, which are essential to combating stigma and improving patient outcomes.

## Editorial

Epidemiological studies over the past five years have shed light on functional neurological disorder (FND)’s significant but underappreciated prevalence. Estimates suggest an annual incidence of 10 to 22 cases per 100,000 people, with a point prevalence exceeding 80 to 140 per 100,000 people, making FND comparable in frequency to conditions such as multiple sclerosis or motor neuron disease [1]. Despite this, under-recognition persists worldwide, exacerbated by limited awareness and diagnostic skills, particularly in low-resource settings.

Disparities in recognition contribute to prolonged patient distress, repeated medical visits, and inappropriate interventions. Global health strategies must prioritize training frontline clinicians to recognize FND confidently, ensuring equitable diagnosis and care irrespective of geography.

FNDs occupy a distinctive position at the interface of neurology and psychiatry, both in their clinical presentation and underlying mechanisms. Neurologically, patients exhibit symptoms such as motor weakness, sensory loss, and non-epileptic seizures, features that mimic organic disease but lack identifiable structural pathology. Psychiatrically, these symptoms often arise in the context of psychological stress, emotional dysregulation, or altered cognitive processing. In this regard, neuroimaging has demonstrated altered functional connectivity in brain regions governing movement, attention, and emotion, highlighting a shared pathophysiological basis that integrates both disciplines [2]. This dual identity challenges traditional categorical thinking and underscores the need for collaborative care models that bring neurologists and mental health professionals into coordinated practice.

Contemporary research has greatly advanced our understanding of the nature and mechanisms of FND. Crucially, the approach to diagnosis has shifted from one of exclusion to one of inclusion. Clinicians are encouraged to make a positive diagnosis of FND by identifying characteristic clinical signs - for example, internal inconsistencies in motor examination findings or symptoms that vary with distraction - rather than merely noting the absence of structural disease [2]. This paradigm change, reflected in the Diagnostic and Statistical Manual of Mental Disorders, Fifth Edition (DSM-5)’s removal of the requirement for a recent psychological stressor, emphasizes that patients’ symptoms are genuinely experienced and reproducibly identifiable. Psychological stressors (such as trauma or life events) are common in the history of FND patients, but notably they are often absent; the lack of an obvious precipitant no longer precludes the diagnosis [2,3].

FND refers to a group of conditions characterized by neurological symptoms, such as motor dysfunction, sensory disturbances, or non-epileptic seizures, that are incompatible with recognized neurological or medical diseases, yet arise from altered brain functioning rather than structural pathology. FND lies at the interface of neurology and psychiatry and is considered a genuine and common cause of neurological disability. According to the DSM-5, the core diagnostic criteria for FND are as follows: one or more symptoms of altered voluntary motor or sensory function, clinical findings provide evidence of incompatibility between the symptom and recognized neurological or medical conditions, the symptom is not better explained by another medical or mental disorder, and the symptom causes clinically significant distress or impairment in social, occupational, or other important areas of functioning or warrants medical evaluation. DSM-5 removed the requirement for a recent psychological stressor or trauma, acknowledging that such factors may be absent in many cases. FND is not a diagnosis of exclusion but one based on positive clinical signs that indicate impaired function of neural systems, often in the absence of damage to those systems [4].

The management of FND has evolved dramatically, with multidisciplinary, patient-centered care now regarded as best practice. Core components include patient education, symptom-specific physiotherapy, and psychological interventions such as cognitive-behavioral therapy (CBT). Explaining the diagnosis in clear, non-blaming terms often reassures patients and lays the groundwork for recovery [2]. The past five years have seen growing research trials testing specific FND interventions. Large multicenter RCTs have examined CBT and physiotherapy-led rehabilitation for FND. The results illustrated progress and complexity: while patients receiving specialized physiotherapy reported more improvement in their symptoms and mental health than those with routine care, objective measures of physical functioning at 12 months showed no significant difference between the groups [3]. This finding highlights both the advances and the remaining challenges in delivering fully effective interventions for all patients. Specialized physiotherapy tailored to address functional motor symptoms uses techniques such as distraction and motor retraining to help patients regain voluntary control. CBT, meanwhile, targets maladaptive thoughts and coexisting anxiety or mood symptoms that can exacerbate functional episodes. When combined, these approaches have shown promising outcomes in multiple trials.

As FND earns its place in mainstream medicine, several challenges and opportunities come to the forefront. Persistent stigma remains one of the greatest barriers to effective FND care. Patients frequently encounter disbelief, both within and outside the medical community, undermining trust and adherence to treatment plans. To counter this, medical education must integrate robust FND training into undergraduate and postgraduate curricula, equipping future practitioners with skills to diagnose and manage FND empathetically [4]. Globally, few dedicated FND clinics exist, creating inequities in access to evidence-based care. Innovative solutions such as telemedicine and digital therapy platforms offer practical avenues to extend specialist support and bridge care gaps through remote consultations and therapy to underserved regions. Digital therapy platforms have emerged as valuable tools in expanding access to FND care, especially in regions lacking specialized services. For example, guided self-management programs delivered via mobile apps or web portals have shown promise in improving symptom understanding and engagement with rehabilitation. Virtual CBT modules tailored to FND can provide structured, evidence-based psychological support remotely, while tele-physiotherapy enables real-time motor retraining with specialist oversight. These innovations reduce geographic and socioeconomic barriers, offering scalable, patient-centered models that extend the reach of multidisciplinary treatment. Health policy must also rise to the occasion: given FND’s prevalence, there is a strong argument for increased funding for FND services and research commensurate with its disease burden. It is encouraging that recent epidemiological data are being used to lobby for this reallocation of resources, highlighting that FND has been under-funded relative to its impact [1]. Research into FND’s pathophysiology and treatment is a fast-moving frontier. Emerging research aims to refine diagnostics and treatments further. Investigations into potential biomarkers, such as functional neuroimaging patterns and neurophysiological signatures, may enable objective confirmation of FND diagnoses in the future. Novel therapeutic strategies, including non-invasive neuromodulation (like transcranial magnetic stimulation) and virtual reality-based rehabilitation, are under exploration, offering hope for improved recovery rates [3].

By its nature, FND spans multiple domains; thus, optimal management requires collaboration between neurologists, psychiatrists/psychologists, physiotherapists, occupational therapists, and others. Reframing FND in modern medicine reflects an important shift toward recognizing it as a treatable condition at the intersection of mind and brain. Recent scientific advances have deepened our understanding of its pathophysiology, clarified its global prevalence, and demonstrated the value of multidisciplinary, personalized care. Continued commitment to clinician education, stigma reduction, and equitable service provision is crucial to ensure that patients worldwide receive the compassionate, evidence-based care they require.
